# Comparative Study on the Transcriptome of Maize Mature Embryos from Two China Elite Hybrids Zhengdan958 and Anyu5

**DOI:** 10.1371/journal.pone.0158028

**Published:** 2016-06-22

**Authors:** Juan Ma, Jingjing Li, Yanyong Cao, Lifeng Wang, Fei Wang, Hao Wang, Huiyong Li

**Affiliations:** Cereal Crops Research Institute, Henan Academy of Agricultural Sciences, Zhengzhou, Henan, China; National Key Laboratory of Crop Genetic Improvement, CHINA

## Abstract

Zhengdan958 and Anyu5 are two elite maize hybrids of China, which manifest similar paternal lines (Chang7-2) but different maternal lines (Zheng58 and Ye478). Zhengdan958 has a 10–15% yield advantage over Anyu5. In this study, we applied digital gene expression technology to analyze transcriptomes of mature embryos from the two hybrids and their parents, aimed to investigate molecular mechanism of heterosis and genetic effects of maternal lines. Results showed that 71.66% and 49.70% of differentially expressed genes exhibited non-additive expression in Zhengdan958 and Anyu5, respectively. The number of non-additive genes involved in abiotic and biotic stress responses in Zhengdan958 was higher than that in Anyu5, which was in agreement with their phenotypic performance. Furthermore, common over-dominance and under-dominance genes (137 and 162, respectively) between the two hybrids focused on plant development and abiotic stress response. Zhengdan958 contained 97 maternal expression-level dominance (maternal-ELD) genes, and the number was higher than that of Anyu5 (45). Common up-regulated maternal-ELD genes were significantly enriched in meristem and shoot development while common down-regulated maternal-ELD genes were involved in pyruvate metabolic process, negative regulation of catalytic activity and response to stress. Therefore, non-additive genes mainly contribute to heterosis in Zhengdan958, including many genes for plant development, abiotic and biotic stress responses. Maternal effects may play important roles in maize heterosis.

## Introduction

Heterosis is the phenomenon that F_1_ hybrids exhibit phenotypes that are superior to those of their parents [1–2]. Plant breeders use heterosis to breed superior varieties in many important crop species. Among them, maize is a typical model crop for studying heterosis. More than 95% of China maize acreage is planted with hybrids. Duvick estimates that maize hybrids exhibit a 15% yield advantage relative to superior open-pollinated varieties and that worldwide heterosis accounts for an additional 55 million metric tons of grain yield annually [3]. Despite the wide use of heterosis in plant breeding, the molecular basis of heterosis is not well understood.

In maize breeding, different inbred lines are divided into stiff stalk heterosis group (SS group) and non-stiff stalk heterosis group (NSS group) on the basis of level of grain yield heterosis. Generally, crosses within heterosis groups show lower grain yield heterosis than crosses between groups. Zheng58, Chang7-2, and Ye478 are the most popular inbred lines in China; Zheng58 and Ye478 belong to SS group (designated as maternal group), and Chang7-2 is an important inbred line of NSS group (designated as paternal group). Chang7-2 exhibits strong hybrid vigor as well as Zheng58 and Ye478. For example, Zheng58 and Chang7-2 are the parental lines of the famous commercial hybrid-Zhengdan958 that is currently the most widely grown variety in China. Ye478 and Chang7-2 are the parental lines of another commercial hybrid-Anyu5, which was widely grown in the 1990s. However, Zhengdan958 shows greater yield, higher planting density, and more stress-tolerance than Anyu5. Lai’s study indicated that Ye478 is one of the parental lines of Zheng58, which inherited 43% of its genomic content from inbred line Ye478 [4]. Thus, genetic difference between maternal lines Zheng58 and Ye478 may account for the difference between Zhengdan958 and Anyu5 in terms of grain yield and resistance.

The availability of high-throughput gene expression profiling technologies currently allows researchers to study the gene expression profile of hybrids relative to the inbred parents [5]. In general, most of these studies focused on characterizing gene expression patterns in single heterotic hybrid compared with the two parents. In this study, we investigated the gene expression profiles of two maize hybrids with common paternal line and different maternal lines using digital gene expression (DGE) technologies, in order to elucidate the molecular mechanism of heterosis and genetic effect of maternal lines.

## Materials and Methods

### Sample collection and RNA extraction

The two maize hybrids Zhengdan985, Anyu5 and their parents Ye478, Zheng58, and Chang7-2 were grown under similar field conditions with three replications. Ten mature seeds of each material were collected at 40 days after fertilization and frozen in liquid nitrogen. Mature embryos were extracted from seeds by hand using scalpels. Three replicate embryo samples of each genotype were mixed equally, and total RNA was extracted with Trizol (Invitrogen).

### Library construction and sequencing

The mRNAs were purified from 6 g of total RNA (300 ng/μl) using poly (T) oligo-attached magnetic beads. After mRNA’s binding, cDNA synthesis was performed. Double strand cDNA was introduced into the cDNA fragment digested by NlaIII endonuclease and the binding fragment containing sequences of CATG site and adjacent poly (A) tail in 3′ end. After the precipitation of 3′ cDNA fragment, Illumina adaptor 1 was added to 5′ end. Both adaptor 1 and CATG site can be recognized by Mme I, which cut at downstream CATG site and produce fragment of 17 bp tags with adaptor 1. Adaptor 2 was added to the 3′ end of these tags after getting rid of fragment with beads in 3′ end. These sequences were prepared for Solexa sequencing. Transcriptome sequencing was performed by sequencing by synthesis method on the Illumina platform at BGI-Shenzhen (China). The data size per sample was 500 Mbp.

### Gene expression classification

Clean-tags were obtained by filtering the adaptor sequences and removing low-quality sequences (containing ambiguous bases). The clean tags were then mapped to the reference genome and genes of the maize available at ftp://ftp.maizesequence.org/pub/maize/release-5b [6]. Only the tags with perfect match or one mismatch were further considered and annotated on the basis of reference genes. The expression level of each gene was estimated by the frequency of clean tags and then normalized to number of transcripts per million clean tags (TPM), which is a standard method that is extensively used in differential gene expression analysis [7]. The expression level of each gene was measured by the normalized number of matched clean tags.

### Identification of differentially expressed genes (DEGs) and gene annotations

For each sequenced library, software package edgeR was used to adjust the read counts by one scaling normalized factor [8]. Differential expression analysis was performed by DEGseq program package for comparisons among samples [9]. Significant DEGs were defined with the Benjamini and Hochberg corrected P-value < 0.05 [10].

Gene ontology (GO) enrichment analyses were conducted according to Benjamini’s study [10]. When the GO database was searched, the GO categorization of all DEGs was displayed as three categories for cellular component, molecular function, and biological process. A GO term was considered significantly enriched if it had a hypergeometric test P < 0.05, a Benjamini and Hochberg (BH) FDR < 0.1.

### Classification of gene expression patterns

Gene expression in hybrids compared to their parents can be classified into additive and non-additive expression. Among non-additive expression, the hybrid gene expression displays a significantly higher level or lower level than both parents, and the two instances have been previously designated above high-parent and below low-parent expression [11] or under-and over-dominance [12]. Moreover, there is such a phenomenon that hybrid gene expression levels which equal that of one parent and differ from that of the other parent in non-additive expression patterns. This phenomenon is termed expression-level dominance (ELD).

## Results

### Transcriptome analysis of Zhengdan958, Anyu5 and their parental lines

In this study, we sequenced five mature embryos DGE libraries from three inbred parents (Zheng58, Ye478 and Chang7-2) and two F_1_ hybrids (Zheng58×Chang7-2 = Zhengdan958; Ye478×Chang7-2 = Anyu5) by massive parallel sequencing on the Illumina platform at BGI-Shenzhen, China. The number of tags for each gene was calculated and normalized to TPM. Given the robustness of subsequent data analysis, only transcripts with more than one TPM were considered as expressed genes. Using these criteria, we obtained 12,859 unique tag-matched genes for Zhengdan958, 13,191 unique tag-matched genes for Anyu5, 13,294 unique tag-matched genes for Zheng58, 12,965 unique tag-matched genes for Ye478, and 12,551 unique tag-matched genes for Chang7-2 (S1 Table). For convenience, we hereafter use AA, BB and CC representing Zheng58, Ye478 and Chang7-2, respectively. Thus, AC indicates Zhengdan958, and BC denotes Anyu5.

### Transcriptome differentiation between two maize hybrids Zhengdan958 and Anyu5

To study the effect of maternal lines and molecular mechanism for heterosis in Zhengdan958 and Anyu5, we analyzed their transcriptomes against paternal inbred line Chang7-2. The two hybrids and Chang7-2 share the common paternal line and different maternal lines. Therefore, it is helpful to have a better understanding of maternal effects on hybrids.

**Fig 1:**
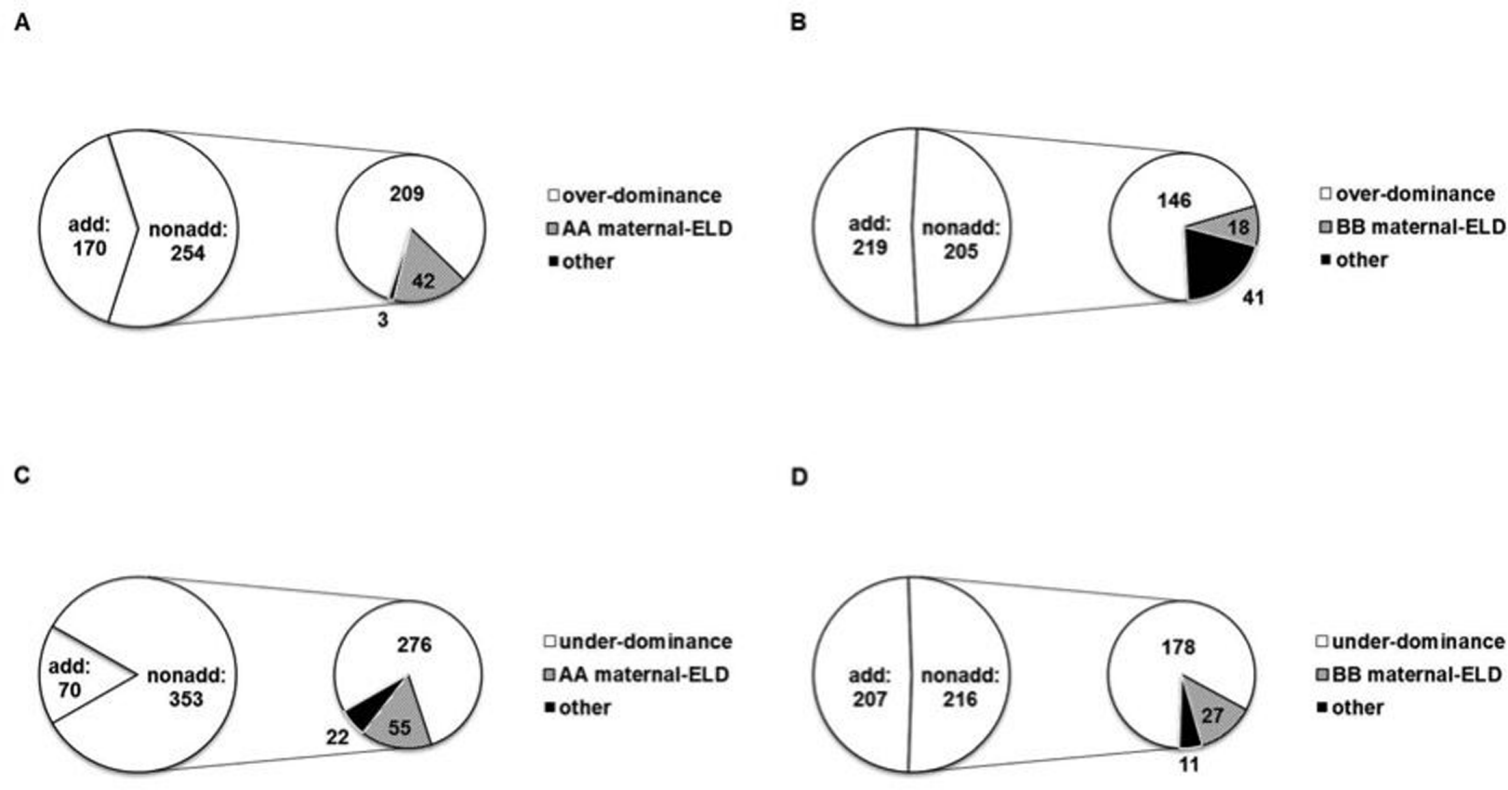
**Pie chart of differentially expressed genes (DEGs) in up-regulated (A, B) and down-regulated (C, D) transcripts in Zhengdan958 (A, C) and Anyu5 (B, D).** add and nonadd represent additive and non-additive genes. maternal-ELD: maternal expression-level dominance. AA and BB represent Zheng58 and Ye478, respectively.

**Fig 2:**
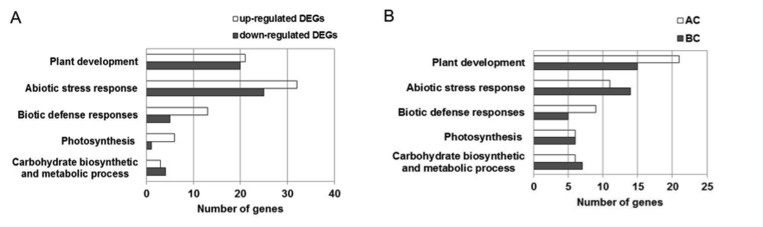
**GO enrichment analysis of up-regulated and down-regulated genes (A) and non-additive genes in Zhengdan958 and Anyu5 (B).** Significantly enriched biological processes associated with carbohydrate biosynthetic and metabolic, biotic defense response, abiotic stress response, photosynthesis related process and plant development were listed (P < 0.05; Benjamini and Hochberg FDR < 0.1). AC and BC denote Zhengdan958 and Anyu5, respectively. DEG: differentially expressed gene.

A total of 847 DEGs among AC, BC and CC transcriptomes were identified. Among them, 424 DEGs (50.06%) were significantly up-regulated (AC>BC>CC), and 423 (49.94%) DEGs were significantly down-regulated (AC<BC<CC) (Fig 1). By GO enrichment analysis, we found up-regulated transcripts contained more DEGs for response to abiotic stress, biotic defense response, and photosynthesis than down-regulated transcripts (Fig 2A). For example, a total of 32 and 25 DEGs associated with response to abiotic stress were significantly enriched in up-regulated transcripts and down-regulated transcripts, respectively. Only three down-regulated genes played roles in response to biotic stress, whereas 12 up-regulated genes were found in this process. In terms of genes associated with photosynthesis, only one gene was found in down-regulated transcripts while six genes were significantly enriched in up-regulated transcripts.

We further classified up-regulated and down-regulated transcripts into non-additive and additive genes. Among 847 genes that were differentially expressed among the two hybrids and their paternal patent, 240 (28.34%) were additive genes while 607 (71.66%) were non-additive genes in Zhengdan958 (Fig 1A and 1C). In Anyu5, 426 and 421 genes displayed additive and non-additive expression, respectively (Fig 1B and 1D).

Subsequently, we pursued possible functions of genes showing non-additive expression (Fig 2B). The results showed non-additive genes in Zhengdan958 and Anyu5 were significantly enriched in carbohydrate biosynthetic and metabolic process and photosynthesis (12 and 13 genes in Zhengdan958 and Anyu5), plant development (21 and 15 genes in Zhengdan958 and Anyu5), abiotic stress response (11 and 14 genes identified in Zhengdan958 and Anyu5), and biotic defense response (nine and four genes in Zhengdan958 and Anyu5). Non-additive expression mainly contributed to heterosis in Zhengdan958, and covered many genes associated with plant development, abiotic and biotic stress responses.

### Over-dominance, under-dominance and maternal expression-level dominance (maternal-ELD) expression pattern in Zhengdan958 and Anyu5

Non-additive genes can be further classified into over-dominance, under-dominance and maternal-ELD genes. Both in Zhengdan958 and Anyu5, we found over-dominance and under-dominance expression accounted for the majority of up-regulated transcripts and down-regulated transcripts, respectively. For up-regulated transcripts, there were 209 (82.28% of 254 non-additive genes) and 146 (71.22% of non-additive 205 genes) over-dominance genes in Zhengdan958 and Anyu5, respectively (Fig 1A and 1B). For down-regulated transcripts, under-dominance genes (78.19%, 276 out of 353 non-additive genes in Zhengdan958; 82.41%, 178 out of 216 non-additive genes in Anyu5) were the most prevalent in the non-additively expressed genes (Fig 1C and 1D).

Both in Zhengdan958 and Anyu5, maternal-dominance genes composed a small portion of non-additive genes in up-regulated or down-regulated transcripts (Fig 1). For Zhengdan958, 16.54% of genes (42 out of 254 up-regulated non-additive genes) represented maternal-ELD while for Anyu5, the percentage of maternal dominance was even lower (8.78%, 18 out of 205 up-regulated non-additive genes). Similar to up-regulated transcripts, a higher portion of maternal-ELD (15.58%, 55 out of 353 non-additive genes) displayed in Zhengdan958 while only 12.50% (27 out of 216 non-additive genes) were Ye478 maternal-ELD in down-regulated transcripts.

**Fig 3:**
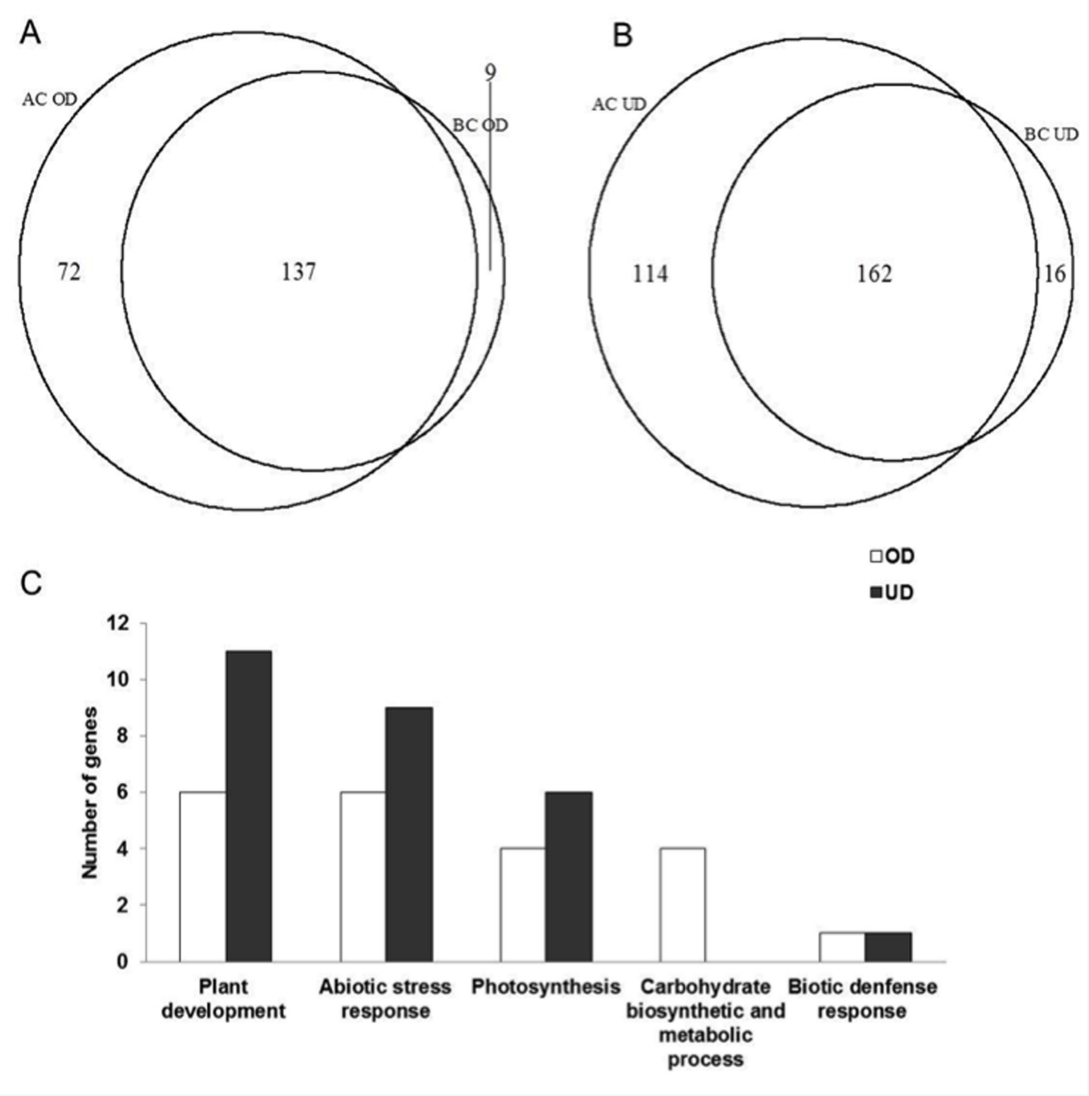
**Venn diagram analysis of over-dominance (OD) (A) and under-dominance (UD) (B) genes in Zhengdan958 and Anyu5, and bar plot of GO enrichment analysis of common over-dominance and under-dominance genes between the two hybrids (C).** AC and BC denote Zhengdan958 and Anyu5, respectively.

**Fig 4:**
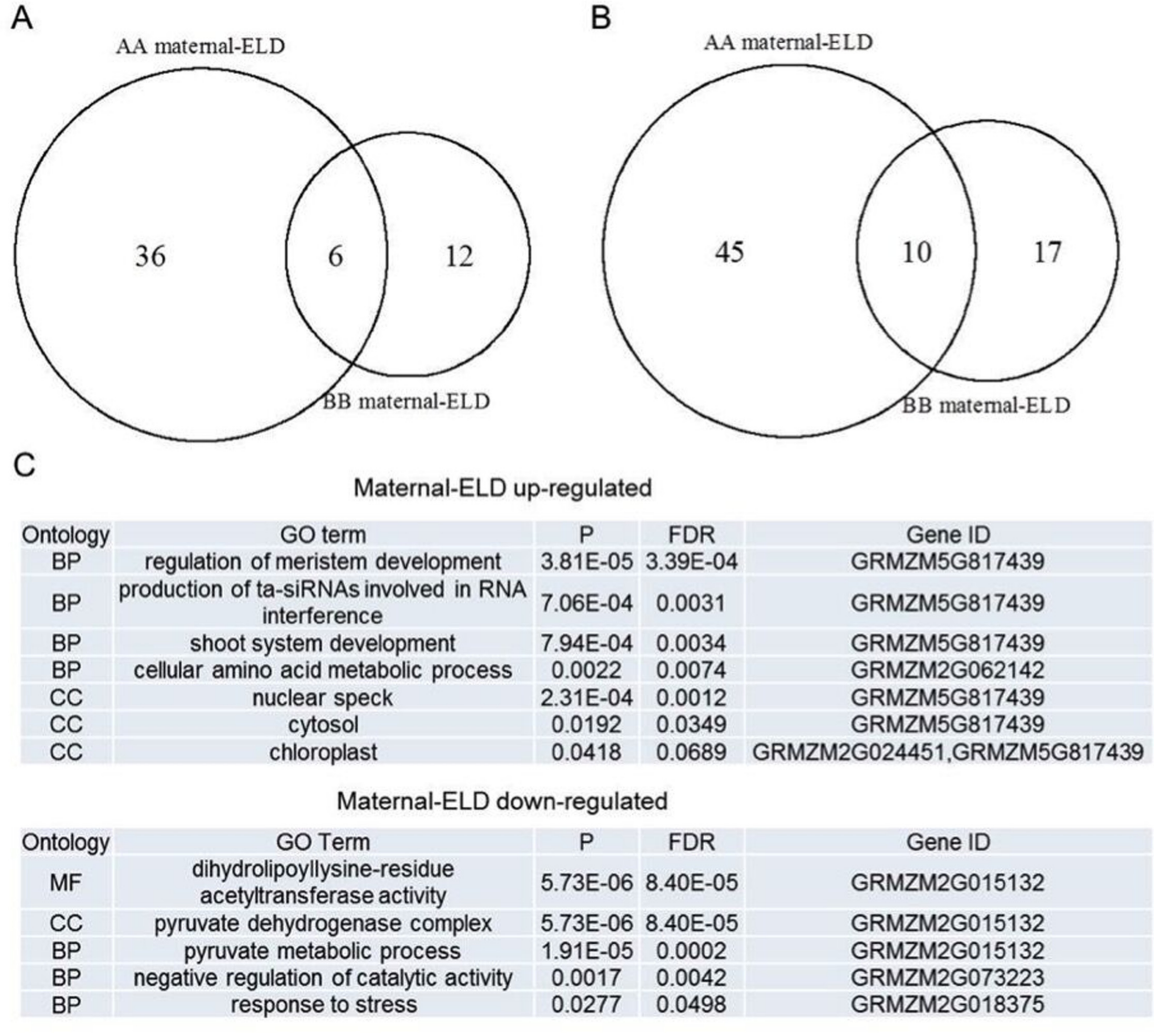
**Venn diagram analysis of maternal expression-level dominance (maternal-ELD) genes in Zhengdan958 (A) and Anyu5 (B), and GO enrichment analysis of common maternal-ELD genes between the two hybrids (C).** AA and BB denote Zheng58 and Ye478, respectively. BP: biological process; CC: cell component; MF: molecular function.

There were 137 over-dominance genes and 162 under-dominance genes that were held in common between Zhengdan958 and Anyu5 (Fig 3A and 3B). We also found the expression of six genes was activated while ten genes were repressed both in Zheng58 and Ye478 maternal-ELD (Fig 4). This indicated that these genes were conserved and important for heterosis.

We firstly investigated GO functional categories of common over-dominance and under-dominance genes. Those common over-dominance genes were significantly enriched in plant development (six genes), abiotic stress response (six genes), photosynthesis (four genes), and carbohydrate biosynthetic and metabolic processes (four genes) (Fig 3C and S2 Table). For common under-dominance genes, there were eleven, nine and six genes associated with plant development, abiotic stress response and carbohydrate metabolic process, respectively (Fig 3C and S3 Table).

The functions of specific over-dominance and under-dominance genes in the two hybrids were analyzed (S4 and S5 Tables). In Zhengdan958, there were seven, four and two over-dominance genes associated with plant development, abiotic stress response and brassinosteroid, respectively. In Anyu5, those specific over-dominance genes were mainly enriched in abiotic stress response. For specific under-dominance genes in Zhengdan958, there were seven genes associated with plant development, four genes for abiotic stress response, two for defense response to bacterium, and two for plant hormone such as indolebutyric acid and abscisic acid. The significant biological process was tetrahydrofolate metabolic process for Anyu5 under-dominance genes.

Analysis of the functional annotations of common genes between Zheng58 and Ye478 maternal dominance were conducted. Up-regulated common genes between Zheng58 and Ye478 maternal-ELD were significantly enriched in regulation of meristem development, shoot system development and cellular amino acid metabolic process (Fig 4C). These genes were in cell components including chloroplast and cytosol. Down-regulated common genes were significantly enriched in the pyruvate metabolic process, negative regulation of catalytic activity and response to stress, and were located in pyruvate dehydrogenase complex (Fig 4C).

We also discovered functional categories of specific maternal-ELD in the two hybrids. GO enrichment analysis indicated that in up-regulated genes, Zheng58 specific maternal-ELD genes were significantly enriched in plant development such as seed germination and leaf morphogenesis, carotenoid biosynthetic process, response to heat and gene silencing. Up-regulated Ye478 maternal-ELD genes were enriched in significant biological processes including photosynthetic and respiratory electron transport chain, defense response to bacterium incompatible interaction and toxin catabolic process (Tables 1 and 2). In down-regulated transcripts, Zheng58 maternal-ELD genes were significantly enriched in cell growth, photorespiration, regulation of ethylene biosynthetic process, response to abscisic acid and tetrahydrofolate metabolic process, while the Ye478 maternal-ELD genes were significantly involved in pollen germination, pollen tube growth, response to phosphate starvation, carotene, xanthophyll and chlorophyll biosynthetic process and epigenetics such as regulation of gene expression epigenetic and chromatin remodeling (Tables 1 and 2). These indicated that Zheng58 and Ye478 specific maternal-ELD genes showed obvious functional distinctions, which may be important players for shaping the difference of heterosis in Zhengdan958 and Anyu5.

**Table 1:** GO enrichment analysis of specific maternal expression-level dominance genes of Zheng58.

GO ID	Biological process	P value	FDR value	Gene ID
**Up-regulated**				
GO:0030154	cell differentiation	0.0011	0.0042	GRMZM2G126128
GO:0009845	seed germination	0.0013	0.0049	GRMZM2G034896
GO:0009965	leaf morphogenesis	0.0042	0.0120	GRMZM2G126128
GO:0016117	carotenoid biosynthetic process	0.0026	0.0078	GRMZM2G126128
GO:0006952	defense response	0.0130	0.0276	GRMZM5G819919
GO:0009408	response to heat	0.0151	0.0313	GRMZM2G034896
GO:0016458	gene silencing	1.62E-04	9.04E-04	GRMZM2G169998
GO:0030001	metal ion transport	0.0088	0.0201	GRMZM2G001803
**Down-regulated**				
GO:0009825	multidimensional cell growth	0.0006	0.0022	GRMZM2G075701
GO:0080167	response to karrikin	0.0099	0.0202	GRMZM6G734131
GO:0009853	photorespiration	0.0012	0.0043	GRMZM2G168281
GO:0010364	regulation of ethylene biosynthetic process	1.14E-05	1.44E-04	GRMZM2G039280
GO:0009737	response to abscisic acid	0.0396	0.0698	GRMZM2G063931
GO:0046653	tetrahydrofolate metabolic process	1.91E-06	8.40E-05	GRMZM2G168281
GO:0006120	mitochondrial electron transport NADH to ubiquinone	1.98E-04	0.0010	GRMZM5G804358

**Table 2:** GO enrichment analysis of specific maternal expression-level dominance genes of Ye478.

GO ID	Biological process	P value	FDR value	Gene ID
**Up-regulated**				
GO:0022904	respiratory electron transport chain	2.86E-05	1.90E-04	GRMZM2G103103
GO:0009767	photosynthetic electron transport chain	7.71E-05	2.79E-04	GRMZM2G103103
GO:0009816	defense response to bacterium incompatible interaction	4.07E-05	1.91E-04	GRMZM2G015889
GO:0009407	toxin catabolic process	3.23E-05	1.90E-04	GRMZM2G102216
**Down-regulated**				
GO:0009846	pollen germination	3.23E-04	0.0011	GRMZM2G162670
GO:0016036	cellular response to phosphate starvation	4.78E-04	0.0015	GRMZM2G377761
GO:0009560	embryo sac egg cell differentiation	5.44E-04	0.0016	GRMZM2G135410
GO:0009860	pollen tube growth	7.15E-04	0.0021	GRMZM2G162670
GO:0016120	carotene biosynthetic process	7.37E-06	3.32E-05	GRMZM2G377761
GO:0016123	xanthophyll biosynthetic process	1.03E-05	4.37E-05	GRMZM2G377761
GO:0015995	chlorophyll biosynthetic process	0.0011	0.0028	GRMZM2G377761
GO:0040029	regulation of gene expression epigenetic	4.92E-06	2.53E-05	GRMZM2G135410
GO:0006338	chromatin remodeling	6.65E-05	2.39E-04	GRMZM2G135410
GO:0046520	sphingoid biosynthetic process	7.37E-06	3.32E-05	GRMZM2G162670

## Discussion

Heterosis is the outstanding performance of hybrids compared to either of their parents. Biological processes during maize mature embryo development after fertilization may be of importance for heterosis. To elucidate genetic effect of heterosis and maternal parents, mature embryos of two commercial maize hybrids Zhengdan958 and Anyu5 were compared using DGE technologies in this study. In a previous study, Zheng58 exhibits 57% difference with Ye478 in genomics [4]. However, our findings indicated that Zheng58 showed 18.17% difference with Ye478 in the mature embryo transcriptome. This result showed that a large portion of differential genes were not expressed in mature embryos. In the present study, several DEGs detected by digital gene expression profiling were already validated by quantitative RT-PCR [13].

### Non-additive genes shapes the China commercial hybrid Zhengdan958 transcriptome

Non-additive expression that significantly differs from the mid-parent value is mainly attributable to trans-regulated interaction in a hybrid. At the transcriptome level, an increasing number of studies in plants showed that non-additive gene expression is quite prevalent in various types of hybrid situations [14–15]. The increase in activity of non-additive genes was associated with increased photosynthesis and determination of cell size and number through development, suggesting that non-additive genes played an important role in biomass heterosis [16]. Meyer et al. [17] proposed that non-additive expression led to higher metabolic activity because of an efficient utilization of resources, and therefore improved performance in hybrids. At the proteome level, Hoecker et al. [18] pointed out that non-additive protein accumulation in young primary roots of maize hybrids might be associated with heterosis manifestation. In addition, many transcripts/e-traits that were controlled many eQTL with opposite allelic effects displayed higher heritability in RIL population than their parents, indicating non-additive genetic variation [19]. Other studies also found a similar number of genes that displayed additive and non-additive expression [20], and the additive gene expression was predominated in heterotic hybrid [21–22]. This discrepancy could be due to the different experimental designs, statistical approaches, different plant materials, different tissues and/or different developmental stages [23]. In this study, non-additive expression patterns were overrepresented in Zhengdan958, and accounted for more than 70% of DEGs. In the hybrid Anyu5, a similar number of additive genes and non-additive genes were identified (426 additive genes and 421 non-additive genes). This data showed the proportional difference of non-additive genes between Zhengdan958 and Anyu5 resulted from the discrepancies of trans-regulated interaction in the two hybrids.

Stress adaption capability is an important factor contributing to heterosis. GO enrichment analysis indicated the total number of non-additive genes involved in abiotic stress response and biotic stress defense in Zhengdan958 were higher than that in Anyu5. This may be in agreement with phenotypic performance that Zhengdan958 shows more adaptation resistance than Anyu5. Therefore, the predominance of non-additive genes in Zhengdan958 may result in yield advantage over Anyu5, and exhibited a trans-regulatory mechanism to act in hybrid mature embryos. A large portion of non-additive genes in this study did not indicate whether this gene expression pattern is solely responsible for heterosis, but it must contribute to hybrid effects to a large extent.

In this study, over-dominance genes accounted for 34.43%-34.68% of non-additive genes in Zhengdan958 and Anyu5. We found about 22.57%-32.54% of common over-dominance genes between the two hybrids, were enriched in abiotic stress responses such as phototropism, photoperiodism flowering and response to desiccation and UV and hyperosmotic salinity response, which were important for maize hybrid vigor (S3 Table). Many studies indicated that over-dominance would lead to heterosis in Arabidopsis [16] and rice [24]. Among these common over-dominance genes, GRMZM2G105400 was significantly enriched in phototropism, anthocyanin accumulation in tissues in response to UV light and response to blue light as well as regulation of hormone levels, cell tip growth, root hair elongation and polysaccharide biosynthetic process. Phototropism allows plants to change the growth direction responding to the location of the light source [25], which is important in maize root and coleoptile development [26–27]. Many studies have shown that three groups of molecules were involved in the mechanism of phototropism in Arabidopsis. The first one was photoreceptor which responded to UV, blue and green light; the second group of molecules were involved in phototropism functions in the signaling pathway activated by photoreceptors, and the third group participated in the process that produced the plant curvature after signals were received [25]. In the third group, studies supported that plant hormone auxin played an important role in differential growth responses in Arabidopsis and maize [28–29]. Therefore, GRMZM2G105400 played a crucial role in phototropism in Zhengdan958 and Anyu5, which was important for hybrid vigor.

### Maternal effects play roles in maize heterosis

Maize hybrids Zhengdan958 and Anyu5 possess the same paternal line but different maternal lines. Thus, the difference in the transcription profiling between Zhengdan958 and Anyu5 may be mainly attribute to maternal lines Zheng58 and Ye478. DNA methylation profiles of the maize endosperm indicated that maternal alleles are more demethylated than paternal alleles, which indicates that maternal alleles are more active than parental alleles [30]. Nodine and Bartel [31] found that maternal parents contribute equally to transcriptome of early embryos as well as paternal lines in Arabidopsis. We found that Zhengdan958 contained more maternal dominance genes than Anyu5 (2.16-fold, 97/45). Significant GO terms indicated that maternal-ELD specific genes were enriched in important biological processes associated with heterosis, and displayed different functions in two hybrids. In addition, common maternal-ELD genes were focus on plant development and response to stress. Therefore, maternal lines may play important roles in maize heterosis by maternal dominance mechanism.

One objective of this study is to elucidate the genetic effects of maternal parents. Investigations of gene functions could be useful to understand maternal effects. Therefore, we discussed functions of several DEGs showing Zheng58 and Ye478 maternal-dominance. Common gene GRMZM2G018375 or thiamine thiazole synthase 1 (*THI1*) was involved in response to stress in Zhengdan958 and Anyu5. Li et al. demonstrated that *THI1* played important roles in both guard cell abscisic acid signaling and drought response in Arabidopsis [32]. Moreover, a large number of studies found that thiamine biosynthesis was up-regulated during plant adaptation responses to abiotic stress such as salt, cold, flood, heat, drought, and osmotic stress [33–36]. And the up-regulation of thiamine biosynthesis under salt and osmotic stress conditions was mediated by abscisic acid at the early seedling stages in Arabidopsis [37]. In this study, gene GRMZM2G063931 involved in response to abscisic acid process were displaying Zheng58 maternal-ELD as well as *THI1*, indicating the regulation of thiamine biosynthesis during plant adaptation responses to abiotic stress may be mediated by abscisic acid in maize mature embryos.

The gene GRMZM2G034896 (Zheng58 maternal-ELD gene) was up-regulated and involved in response to heat and seed germination in this study. Its homolog in Arabidopsis, respiratory burst oxidase homolog protein B *RBOHB*, also played essential roles in plant development such as seed germination and seedling elongation development [38] as well as response to abiotic stimuli and pathogen defense [39]. The Ye478 maternal-ELD gene GRMZM2G015889 was activated and associated with defense response to bacterium incompatible interaction. This is consistent with the function of its homolog serine/threonine-protein kinase *PBS1* that was required in specific recognition of the bacterial protein in Arabidopsis [40]. Lee and Kim also found that *OsPBL1* is involved in antiviral defense signaling pathways in rice [41]. An overexpression of the two DEGs in mature hybrid embryos may prompt stress adaption capability in seed development and possible roles for hybrid vigor.

Two DEGs (GRMZM2G063931 and GRMZM2G039280) associated with plant hormone displayed maternal-ELD of Zheng58, and were in a down-regulated state. GRMZM2G063931 (ubiquitin-conjugating enzyme E2) was involved in response to abscisic acid. Ubiquitin plays important roles in maintaining cellular homeostasis and enables effective adaptation to environmental changes. Seo et al. reported that the role of ubiquitination was associated with hormonal signaling [42]. Therefore, the maternal-ELD gene GRMZM2G063931 may regulate plant response to abiotic stress through hormone abscisic acid mediated by ubiquitination in Zhengdan958. GRMZM2G039280 (ethylene-overproduction protein 1) was significantly involved in regulation of ethylene biosynthetic process, and played roles in response to drought as well in this study. Moreover, Du et al. reported that *OsETOL1*, the homolog of GRMZM2G039280, played distinct roles in drought and submergence tolerance by modulating ethylene production and energy metabolism in rice [43]. When ethylene biosynthesis pathway is repressed, it may induce changes in a wide range of developmental processes and adaptation responses [44].

In addition, the gene GRMZM2G135410 coding for chromatin remodeling and regulation of gene expression epigenetic proteins were expressed in the way of Ye478 maternal-ELD pattern, indicating a contribution to epigenetic regulation of hybrids. One gene GRMZM2G169998 showing the Zheng58 maternal-ELD pattern was also significantly involved in epigenetic modification process gene silencing. A recent study revealed that epigenetics might be helpful and complementary to conventional genetic explanations of the molecular mechanism of heterosis [45]. This might be true for embryo development after fertilization, because the interaction of the different parental genomes has to be coordinated and adjusted for generating heterosis [46].

In summary, the analysis of gene expression during mature seed embryos manifestation as well as the analysis of non-additive genes may contribute to a better understanding of the molecular networks regulating the heterosis and effect of maternal lines in Zhengdan958 and Anyu5.

## Supporting Information

S1 Table Expressed genes in Zhengdan958, Anyu5, Zheng58, Ye478 and Chang7-2. (XLSX)

S2 Table GO enrichment analysis of common over-dominance genes between Zhengdan958 and Anyu5. (XLSX)

S3 Table GO enrichment analysis of common under-dominance genes between Zhengdan958 and Anyu5. (XLSX)

S4 Table GO enrichment analysis of specific over-dominance genes in Zhengdan958 and Anyu5. (XLSX)

S5 Table GO enrichment analysis of specific under-dominance genes in Zhengdan958 and Anyu5. (XLSX)
